# BioEGRE: a linguistic topology enhanced method for biomedical relation extraction based on BioELECTRA and graph pointer neural network

**DOI:** 10.1186/s12859-023-05601-9

**Published:** 2023-12-19

**Authors:** Xiangwen Zheng, Xuanze Wang, Xiaowei Luo, Fan Tong, Dongsheng Zhao

**Affiliations:** Academy of Military Medical Sciences, Beijing, 100039 China

**Keywords:** Biomedical relation extraction, Text mining, BioELECTRA, SciSpaCy, Graph pointer neural network, Topological features

## Abstract

**Background:**

Automatic and accurate extraction of diverse biomedical relations from literature is a crucial component of bio-medical text mining. Currently, stacking various classification networks on pre-trained language models to perform fine-tuning is a common framework to end-to-end solve the biomedical relation extraction (BioRE) problem. However, the sequence-based pre-trained language models underutilize the graphical topology of language to some extent. In addition, sequence-oriented deep neural networks have limitations in processing graphical features.

**Results:**

In this paper, we propose a novel method for sentence-level BioRE task, BioEGRE (**BioE**LECTRA and **G**raph pointer neural net-work for **R**elation **E**xtraction), aimed at leveraging the linguistic topological features. First, the biomedical literature is preprocessed to retain sentences involving pre-defined entity pairs. Secondly, SciSpaCy is employed to conduct dependency parsing; sentences are modeled as graphs based on the parsing results; BioELECTRA is utilized to generate token-level representations, which are modeled as attributes of nodes in the sentence graphs; a graph pointer neural network layer is employed to select the most relevant multi-hop neighbors to optimize representations; a fully-connected neural network layer is employed to generate the sentence-level representation. Finally, the Softmax function is employed to calculate the probabilities. Our proposed method is evaluated on three BioRE tasks: a multi-class (CHEMPROT) and two binary tasks (GAD and EU-ADR). The results show that our method achieves F1-scores of 79.97% (CHEMPROT), 83.31% (GAD), and 83.51% (EU-ADR), surpassing the performance of existing state-of-the-art models.

**Conclusion:**

The experimental results on 3 biomedical benchmark datasets demonstrate the effectiveness and generalization of BioEGRE, which indicates that linguistic topology and a graph pointer neural network layer explicitly improve performance for BioRE tasks.

## Background

Biomedical relation extraction (BioRE) [1, 2] is a subtask of relation extraction (RE) [3, 4], which is to determine whether a pre-fetched entity pair in a sentence has a biomedical relationship. BioRE tasks can be categorized into binary and multi-class RE tasks. Compared to a binary task, a multi-class task is more challenging and rewarding, which requires not only deducing whether a relation exists between the entities but also analyzing the specific relation type. Automatic and accurate extraction of relations in biomedical literature is a crucial step in transforming unstructured biomedical knowledge into structured forms, which has the potential to assist researchers in tracking and summarizing biomedical knowledge contained in an extensive range of scientific literature [5].

Recently, propelled by advancements in deep learning [6], models based on deep neural networks (DNNs) have emerged as trustworthy tools for addressing natural language processing (NLP) tasks. DNN-based methods allow for end-to-end extraction of relations in biomedical literature, thus decreasing labor costs and enhancing performance [7]. To tackle the problem of insufficient high-quality and large-scale annotated data, pre-trained language models (LMs), such as Word2Vec [8], ELMo [9], BERT [10], and ELECTRA [11], acquire distributed representations of tokens from large unannotated corpora using a self-supervised learning strategy and perform fine-tuning for a specific downstream task. The pre-training strategy has also been applied in the biomedical field, leading to the development of biomedical pre-trained LMs such as BioWord2Vec [12], BioELMo [13], BioBERT [14], and BioELECTRA [15], which have been successively proposed and applied to the BioRE task, achieving state-of-the-art (SOTA) performance.

**Fig. 1:**
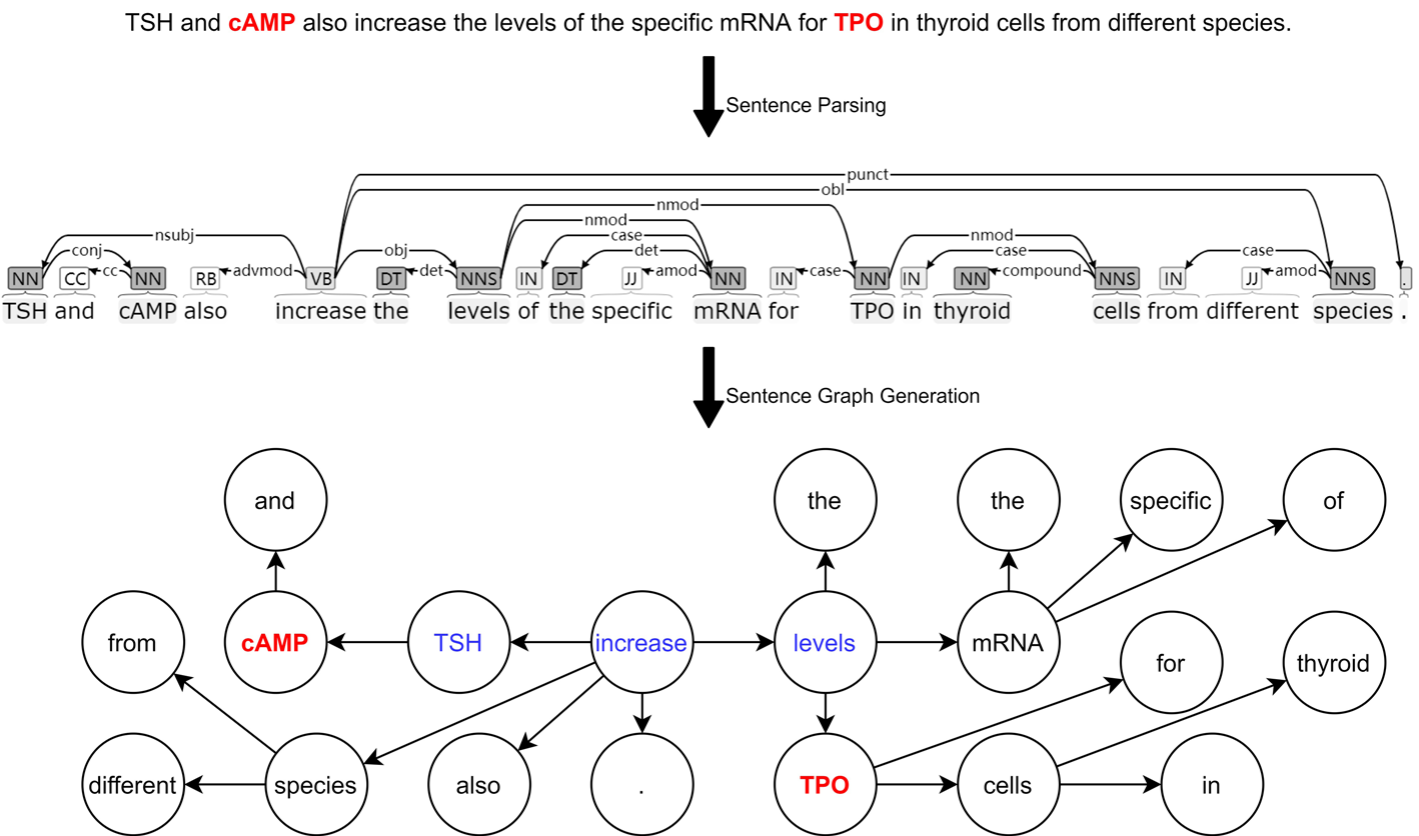
An example of sequential and graphical modeling of a sentence. In the sequential model, the distance of a target entity pair (cAMP and TPO) is 9, while in the graphical model, the distance is shortened to 3. In comparison, modeling the topology of a sentence through a graph can better define the distance between words, which benefits the implementation of NLP downstream tasks to some extent

However, the aforementioned methods primarily focus on semantic information at the sequence level, overlooking the potential of linguistic topology features and geometrical topological features derived from dependency parsing results. A sentence not only contains a subject, a predicate, and objects, reflecting the main semantics, but also includes modifiers, such as attributes, adverbials, complements [16, 17]. As depicted in Fig. 1, the presence of modifiers causes key components to be separated, thereby highlighting the limitations of modeling sentences through sequences. Given that previous studies on biomedical named entity recognition have successfully employed graphical sentence models, leveraging pre-trained LMs and graph neural networks, and resulting in state-of-the-art (SOTA) performance [18–20], it is reasonable to consider applying a similar framework to the BioRE and other BioNLP tasks.

In this paper, drawing inspiration from the prior knowledge of linguistic topology, we adopt a graph-based approach to model sentences and propose a novel method for sentence-level BioRE, BioEGRE (BioELECTRA and Graph pointer neural network for Relation Extraction). First, the biomedical literature is preprocessed to retain sentences involving pre-defined entity pairs. Secondly, SciSpaCy is employed to conduct dependency parsing; sentences are modeled as graphs based on the parsing results; BioELECTRA is utilized to generate token-level representations, which are modeled as attributes of nodes in the sentence graphs; a graph pointer neural network layer is employed to select the most relevant multi-hop neighbors to optimize representations; a fully-connected neural network layer is employed to generate the sentence-level representation. Finally, the Softmax function is employed to calculate the probabilities obtain the results. BioEGRE is first evaluated on the CHEMPROT, which is a high-quality corpus specifically designed for the extraction of chemical-protein interactions (CPI) in the field of biomedicine, representing a multi-class BioRE task. In addition, we evaluate the performance of BioEGRE on binary BioRE tasks focused on gene-disease (using the GAD corpus) and disease-target (using the EU-ADR corpus) extraction. The experimental results demonstrate that BioEGRE achieves an F1-score of 79.97% (CHEMPROT), 83.31% (GAD), and 83.51% (EU-ADR) in the aforementioned BioRE tasks, surpassing current SOTA models and highlighting both the high performance and generalizability of BioEGRE.

Our contributions are summarized as follows:

1. A novel method for BioRE tasks, which models a sentence as a graph, obtains contextual features via BioELECTRA and fuses linguistic topological features through a graph pointer neural network (GPNN) layer.
2. Extensive experiments demonstrate that the proposed method consistently outperforms current SOTA methods, highlighting the significant improvement achieved by incorporating linguistic topological features and a GPNN layer for various BioRE tasks. The remainder of this paper is organized as follows. In chapter II, related work is covered. In chapter III, the proposed model is introduced in details. And in chapter IV, the designed experiments, corresponding results, and discussions are presented. Finally, the paper is concluded in chapter V.

## Related work

In this chapter, we first introduce the pre-trained LM based methods for BioRE. Then, we describe the preliminary knowledge of our method, including topological features of the language and the graph neural network.

### Pre-trained LM based methods for BioRE

Pre-trained LMs, including CoVe [21], ELMo [9], GPT [22] and BERT [10], have the ability to adapt distributed representations based on contextual information. There are also many derivatives of BERT, including BioBERT [14], SciBERT [23], PubMedBERT [24], and ClinicalBERT [25]. Using BERT-based pre-trained LMs has become the paradigm of BioRE in recent years. As for the problem of CPI extraction, [26] utilizes Gaussian probability distribution to introduce biomedical prior knowledge based on BERT. Sun et al. [5] adopts a capsule network and leverages the attention mechanism based on BERT. Zuo and Zhang [27] employs a span-based BERT along with a multi-task learning strategy to jointly extract biomedical entities and relationships. Moreover, [28] and [14] performs pre-training on extra biomedical data based on the initialized weights of BERT respectively, which can be applied to various BioRE tasks.

ELECTRA [11], which is based on the generative adversarial strategy, represents another type of pre-trained language model, which is comprised of a transformer-based generator and discriminator, and achieves SOTA results with minimal computation. As a derivative of ELECTRA, BioELECTRA [15] has achieved SOTA results on various BioRE tasks.

Recently, GPT-based models such as ChatGPT and GPT-4 have revolutionized the approach to general NLP. GPT, a Transformer-based pre-trained language model designed for general domains. The powerful generative ability of GPT-based LMs enhances their generalization and intelligence. Apart from BERT-based pre-trained LMs, GPT-based LMs have also been applied to BioRE tasks. BioGPT [29], which is pre-trained with biomedical data based on GPT-2 architecture, tackles BioRE by generating relational triplets based on well-defined prompts. BioGPT demonstrates the ability to conduct BioRE, whether the relationship is pre-defined or not, thereby introducing new possibilities for this task. While GPT-based models exhibit strong performance in certain generation NLP tasks such as translation, writing, and question-answering, they currently do not match the performance of BERT-based LMs in biomedical RE tasks due to the absence of well-defined, domain-specific prompts.

### Topology of language

The part of grammar that presents a speaker’s knowledge of sentences and their structures is called syntax [30], which is the pattern of language. Syntax is one of the essential objects and the crucial characteristics of downstream tasks in the NLP field [31]. The syntax of a sentence determines its topology, which can be represented as a graph (or a tree) [18]. In comparison to sequences, graphs offer a more comprehensive representation of the semantic relationships between words. So far, the topological features of language have been utilized in RE tasks. Miwa and Bansal [32] constructs a Bi-TreeLSTM layer for sentence dependencies to improve performance. In addition, [33] introduces the shortest dependency path (SDP) between the target entity pair by leveraging dependency relationships, which incorporates a Bi-LSTM layer to identify adverse drug reaction (ADR) knowledge in texts.

Sentence parsing is a technique used to extract topological features from text. Currently, there are several open-source NLP tools available for performing automatic and accurate parsing, including NLTK [34], StandfordNLP [35], and SpaCy [36]. Among them, SpaCy is a fast, powerful, lightweight NLP tool that can handle multiple languages, of which the functions include tokenizer, parser, and tagger. SciSpaCy [37] is an extension of SpaCy that inherits its diverse range of functionalities. Moreover, SciSpaCy is specifically trained on biomedical literature, rendering it more tailored to the unique characteristics of the biomedical field. SciSpaCy achieves F1-score of 98.86% on parsing tasks, demonstrating that it is a reliable biomedical NLP tool.

### Graph neural networks (GNNs)

Recently, the advancement of data analysis has led to the modeling of non-Euclidean data in more complex structures such as trees and graphs, as opposed to simple linear models. Consequently, there has been an increasing focus on leveraging deep learning techniques for analyzing graph-based data, giving rise to the emergence of graph neural networks (GNNs) [38].

Graph convolutional networks (GCNs) [39], graph attention networks (GATs) [40], and graph pointer neural networks (GPNNs) [41] are outstanding representatives of GNNs. Among them, GCN [39] uses a kernel to associate nodes and their neighbors, which obtains only local features. Different from the fixed kernel of GCN, GAT [40] employs a masked self-attention mechanism that dynamically computes weights based on the topology of the graph to obtain a more accurate distributed representation for a node. However, in a heterogeneous graph, the distance between related nodes can be considerable, which implies that the aforementioned GNNs fail to capture features entirely and introduce noises to some extent. To solve the above problems, Yang et al. propose the GPNN [41], which comprises a multi-hop node sequence sampler and a graph pointer generator, which generates an ordered neighbor node sequence according to the degree of correlation to a central node, and an optimized representation for the node according to features from the ranked neighbors. The distributed representation can be used for downstream tasks such as node classification.

## Methodology

### Problem definition

In the field of machine learning, BioRE can be considered as a classification problem, which can be formulated as follows: Given a collection of sentences that include a pre-defined entity pair, the task is to predict the relation types by calculating the conditional probability of sentences belonging to the respective pre-defined labels.

BioRE is mainly divided into binary RE and multi-class RE. Binary RE aims to identify whether a pre-fetched entity pair has a semantic relationship in a sentence ignoring the semantic type, which can be implemented relative easily. Multi-class RE tasks require determining both the presence of a relationship in a sentence and classifying it into a specific semantic type, which is a more challenging and valuable research objective. Therefore, we choose the CPI extraction, which is a multi-class RE task, as an application case to validate the effectiveness of the proposed method.

The objective of CPI extraction is to identify whether a candidate sentence contains a relationship between a chemical-protein pair. If such a relationship exists, a specific chemical-protein relation (CPR) type also need to be classified. The CHEMPROT [42] corpus is a manually labeled corpus for CPI extraction, involving 5 pre-defined CPR types for evaluation, including CPR:3, CPR:4, CPR:5, CPR:6 and CPR:9. Therefore, we formulate CPI extraction task as a six-classification task, including the above five types of pre-defined positive CPRs, while CPR:1, CPR:2, CPR:7, CPR:8, CPR:10, and False to represent negative samples. Table 1 describes definitions of the pre-defined CPRs in CHEMPROT.

**Table 1:** Predefined CPRs in CHEMPROT

Group	Evaluation	CHEMPROT relations belonging to this group
CPR:1	N	PART_OF
CPR:2	N	REGULATOR|DIRECT_REGULATOR|INDIRECT_REGULATOR
CPR:3	Y	UPREGULATOR|ACTIVATOR|INDIRECT_UPREGULATOR
CPR:4	Y	DOWNREGULATOR|INHIBITOR|INDIRECT_DOWNREGULATOR
CPR:5	Y	AGONIST|AGONIST-ACTIVATOR|AGONIST-INHIBITOR
CPR:6	Y	ANTAGONIST
CPR:7	N	MODULATOR|MODULATOR-ACTIVATOR|MODULATOR-INHIBITOR
CPR:8	N	COFACTOR
CPR:9	Y	SUBSTRATE|PRODUCT_OF|SUBSTRATE_PRODUCT_OF
CPR:10	N	NOT

The formal definition of the above six-classification task is as follows. Given a set of candidate sentences $S=\left\{{s}_{1}, {s}_{2}, \dots , {s}_{n}\right\}$, each ${s}_{i}\in S$ contains a chemical-protein pair, and $n$ is the number of sentences. The goal is to infer the relation type of ${s}_{i}$ by calculating the conditional probability $P({r}_{j}|{s}_{i})$ of ${s}_{i}$ falling into ${r}_{j}\in \{CPR:3, CPR:4, CPR:5, CPR:6, CPR:9, False\}$ label.

### Overall architecture

**Fig. 2:**
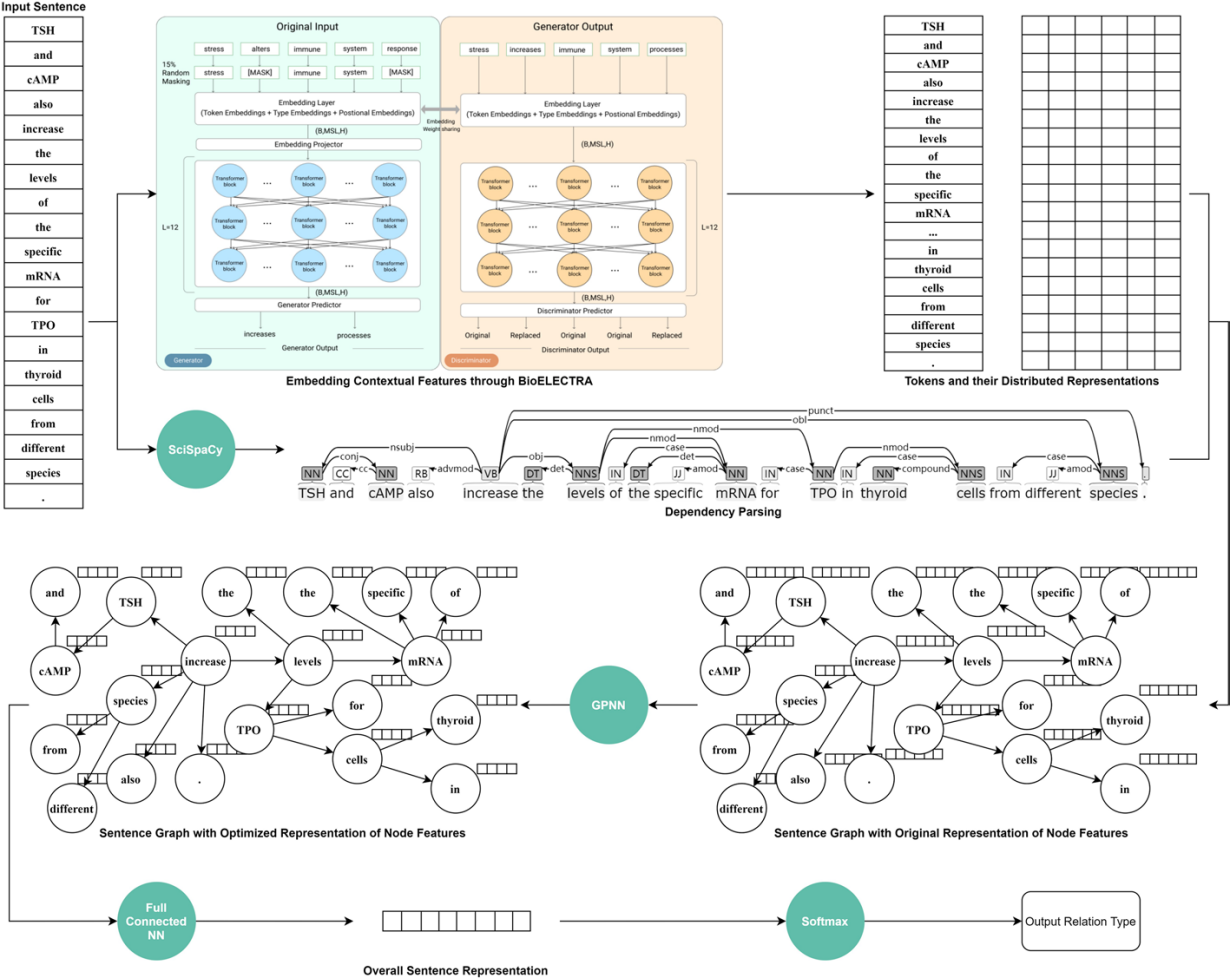
The overall architecture of BioEGRE

BioEGRE consists of three components: an input, a representation, and an output module. The overall architecture of BioEGRE is illustrated in Fig. 2.

**Fig. 3:**
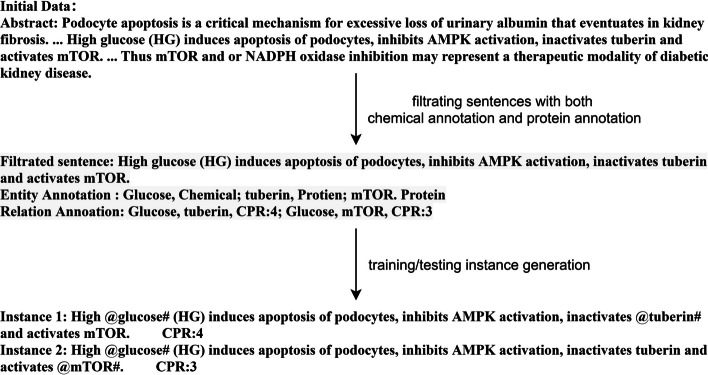
Processing procedure of the input module

First, in the input module, an NLP tool SciSpaCy is used to separate sentences and words; entities in sentences are located according to the original corpus; sentences containing a pre-labeled entity pair (eg. chemical-protein) are screened out as follow-up subjects; entities are marked with a ‘@’ at the beginning and a ‘#’ at the end to help to get the location of entities in sentences. If a sentence contains multiple pre-labeled entity pairs, it is mapped to several instances, each of which contains only one pair of pre-fetched entities. As shown in Fig. 3, a sentence with 1 chemical mention and 2 protein mentions is acquired. The input module generates 2 instances based on the sentence. One is oriented to Glucose and tuberin, labeled as CPR:4, and the other is oriented to Glucose and mTOR, labeled as CPR:3.

Second, in the representation module, BioELECTRA is used to obtain the distributed representation of contextual features for tokens in a sentence; SciSpaCy is utilized to get topological features to construct the sentence graph; a GPNN layer is employed to optimize representations of nodes in the sentence graph; and a full-connected neural network layer is used to generate the overall sentence-level representation.

Finally, in the output module, considering it is a multi-classification task, the Softmax function is implemented to compute the probability distribution for each label. Based on the optimized sentence-level representation, the CPR type is determined.

### Construction of sentence graph

A graph is a data structure of computer science to instantiate the undirected and directed graph in graph theory of mathematics [43], which consists of nodes and edges. Let $G=(V, E)$ be a graph, of which a node set is $V$ and an edge set is $E$. Considering dependency and topological features of language, a sentence can be modeled as an undirected graph.

**Figure:**
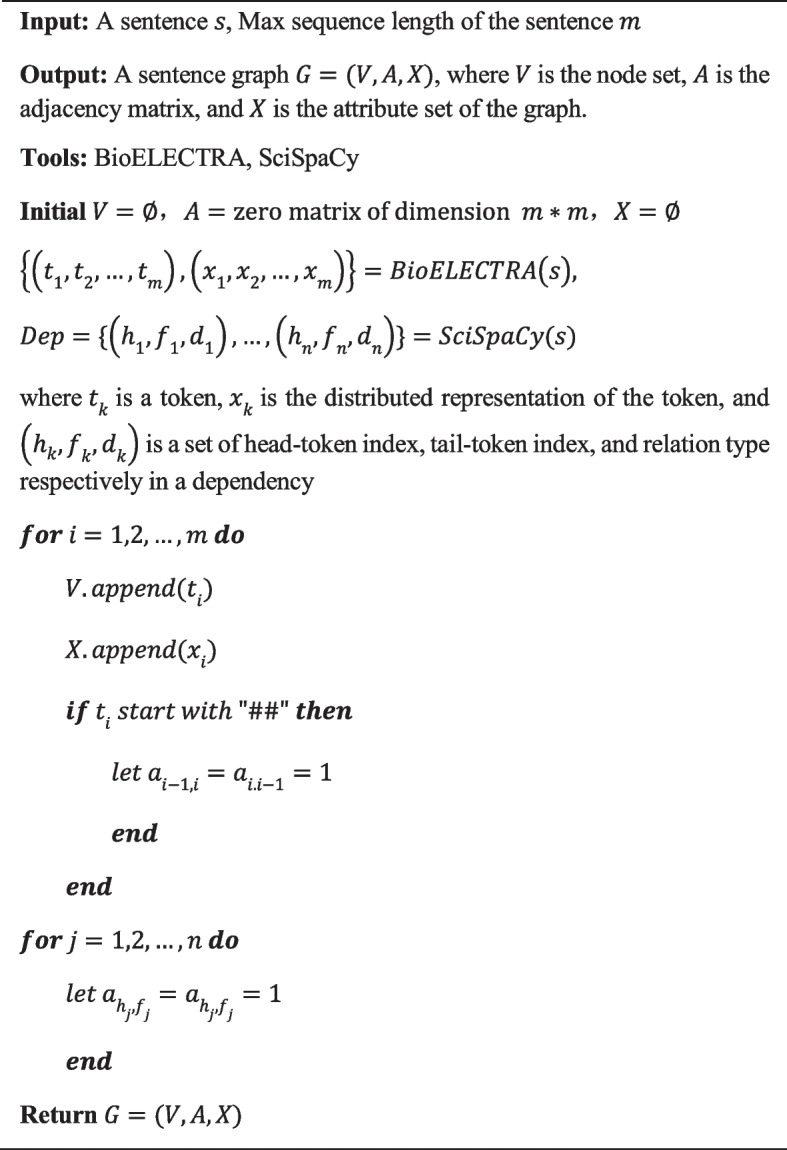
**Algorithm 1: Generation of a sentence graph**

BioELECTRA is used to tokenize and encode sentences and obtain the token-level contextual features, and SciSpacy is used to parse sentences to get the dependencies. Note that BioELECTRA splits obscure words into word-pieces while tokenization to avoid the OOV problem (for example, 'tacrine' is split into ['tac',' ##rine'] by the tokenizer of BioELECTRA). For a sentence ${s}_{i}\in S$, we get a cluster of tokens, ${s}_{i}=({t}_{1}^{i}, {t}_{2}^{i}, \dots , {t}_{m}^{i})$, along with its distributed representation ${X}_{i}=({x}_{1}^{i}, {x}_{2}^{i}, \dots , {x}_{m}^{i})$, where $m$ represents the number of tokens. The detailed procedure for constructing a sentence graph is illustrated in Algorithm 1. In the graph, each token is modaled as a node, and edges are defined based on the following rules: if there is a dependency between two words, the corresponding nodes of the 2 words are connected by an edge. Especially, if a word is divided into word-pieces by the tokenizer of BioELECTRA, the first piece of the word is linked to the other word which has a dependency with it, and the pieces of the word are also linked in order. The token-level representation is modeled as the attribute of the corresponding node. In this way, a graph ${G}_{i}=({V}_{i}, {A}_{i}, {X}_{i})$ for the sentence ${s}_{i}$ is generated, in which ${V}_{i}, {A}_{i}, {X}_{i}$ are respectively a node set, an adjacency matrix, and an attribute set to describe the sentence graph. Adjacency matrix ${A}_{i}$ is a symmetric matrix of $m*m$, and the element is defined as the following formula.

1 $$\begin{array}{*{20}c} {a_{p,q}^{i} \in A_{i} = \left\{ {\begin{array}{*{20}l} 1 \hfill & {if\;t_{p} \;and\;t_{q} are\;neighbors} \hfill \\ 0 \hfill & {else} \hfill \\ \end{array} 0 \le p,q \le m} \right.} \\ \end{array}$$

### Generation of sentence representation

In this section, we provide a detailed explanation of the process for generating the sentence-level distributed representation using a GPNN layer and a fully-connected neural network layer.

**Figure:**
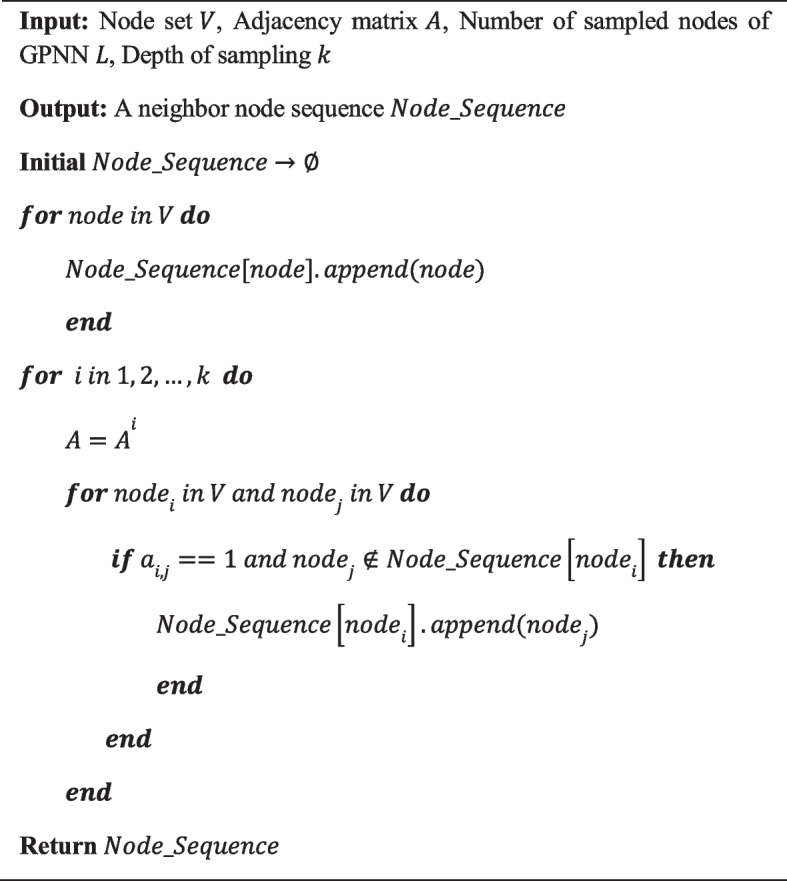
**Algorithm 2: Node sequence sampling based on multi-hop strategy**

The GPNN consists of a multi-hop node sequence sampler and a graph pointer generator. For a graph ${G}_{i}$, a neighbor sequence for each node is generated by using a multi-hop node sequence sampling strategy. The process of generating the neighbor sequence for a node is described in Algorithm 2. For each node in ${G}_{i}$, neighbors are sampled from 1-hop to $k$-hop with the Breath-First-Search (BFS) strategy. Nodes in the neighbor sequence are sorted according to the hops, and neighbors in the same hops are in random order. In theory, the sampling depth $k$ can be set to sample all the nodes in a graph, which guarantees the sampler to capture long-distance features. Considering that some nodes have too many neighbors, we also set a number of sampled nodes of GPNN $L$ to stop sampling. Hyperparameters $k$ and $L$ can be adjusted flexibly to meet the requirements of different applications.

First, in the graph pointer generator module, a GCN layer is applied to ${G}_{i}$ to obtain local features.

2 $$\begin{array}{*{20}c} {\widehat{{X_{i} }} = GCN\left( {X_{i} } \right) X_{i} \in R^{{m{*}d_{1} }} , \widehat{{X_{i} }} \in R^{{m{*}d_{2} }} } \\ \end{array}$$

where ${X}_{i}$ is the original distributed representation of the node, $\widehat{{X}_{i}}$ fuses local features in the graph, $m$ is the number of nodes, ${d}_{1}$ is the dimension of original representation, and $GCN$ is graph convolution calculation.

A vanilla GCN layer [39] (as shown in formula 3) is utilized to implement the above calculation, and the input features are embedded into a vector space with the dimension of ${d}_{2}$.

3 $$\begin{array}{*{20}c} {x_{p}^{i,t} = \sigma \left( {W\mathop \sum \limits_{{q \in N_{1} \left( p \right)}} \frac{1}{{\sqrt {\hat{d}_{p} \hat{d}_{q} } }}x_{q}^{i,t - 1} } \right)} \\ \end{array}$$

where $\sigma \left(\bullet \right)$ is an activate function, $W$ is a trainable matrix, ${N}_{1}(p)$ is the cluster of 1-hop neighbors for $p$, $t$ is the hidden layer propagation step, ${\widehat{d}}_{p}$ is the degree of $p$, and $1/\sqrt{{\widehat{d}}_{p}{\widehat{d}}_{q}}$ represents the weight between $p$ and $q$.

Second, an LSTM-based sequence-to-sequence framework is used to screen out related nodes to the central node from the neighbor sequence and sort them by correlation. Specifically, two separate LSTM layers are applied, one as the encoder and the other as the decoder. For each node in the graph, the sampled neighbors $\{{n}_{1}, {n}_{2}, \dots , {n}_{L}\}$ and its representation $\{{\widehat{x}}_{{n}_{1}}, {\widehat{x}}_{{n}_{2}}, \dots , {\widehat{x}}_{{n}_{L}}\}$ are input into the encoder. And on the ${t}^{th}$ input, the representation of hidden layer representation is calculated as follows.

4 $$\begin{array}{*{20}c} {e_{t} = \tanh \left( {W\left[ {e_{t - 1} ,\hat{x}_{{n_{t} }} } \right]} \right) 0 \le t \le L} \\ \end{array}$$

where ${e}_{0}$ is initialized as 0, $tanh\left(\bullet \right)$ is an activate function, $W$ is a trainable matrix, and $L$ the number of sampled nodes of GPNN. After the above steps, we obtain $E=\{{e}_{1}, {e}_{2},\dots , {e}_{L}\}$, which reflects the overall feature of nodes in the neighbor sequence.

Then, in the decoder, the representation of hidden layer representation is calculated as formula 5.

5 $$\begin{array}{*{20}c} {d_{t} = \tanh \left( {W\left[ {d_{t - 1} ,\hat{x}_{{c_{t - 1} }} } \right]} \right) 0 \le t \le M} \\ \end{array}$$

where ${c}_{t-1}$ is the index of the selected node at the time $t-1$, ${c}_{0}$ is the predefined label [start], and $M$ is the number of screened out nodes of GPNN.

Third, using the hidden representation of the encoder and decoder, the conditional probability is calculated to rank nodes.

6 $$\begin{array}{*{20}c} {P\left( {c_{p} {|}c_{1} ,c_{2} , \ldots ,c_{p - 1} ,\hat{x}_{{n_{1} }} ,{ }\hat{x}_{{n_{2} }} ,{ } \ldots ,{ }\hat{x}_{{n_{L} }} } \right) = softmax\left( {v^{T} \tanh \left( {W_{1} e_{q} + W_{2} d_{p} } \right)} \right)} \\ \end{array}$$

where $0\le q\le L, 0\le p\le M$, $softmax(\bullet )$ is a normalized function, $v,{W}_{1},{W}_{2}$ are trainable weights, and $T$ is a transpose operation. After that, the output sequence is acquired.

7 $$\begin{array}{*{20}c} {output = \left\{ {\hat{x}_{{c_{1} }} ,{ }\hat{x}_{{c_{2} }} ,{ } \ldots ,{ }\hat{x}_{{c_{m} }} } \right\}} \\ \end{array}$$

Fourth, based on the selection and ranking of the neighbors, a 1D GCN layer is employed to extract and integrate topological features, generating the optimized representation of global features for the central node.

8 $$\begin{array}{*{20}c} {Z = Aggregation\left( {ConV\left( {ouptut} \right)} \right)} \\ \end{array}$$

where $Aggregation(\bullet )$ is an aggregation operation, and $ConV(\bullet )$ denotes the 1D convolution operation.

Fifth, during generating the final token-level representation, considering that the emphasis of $X$, $\widehat{X}$ and $Z$ is different but may have couplings, a full-connected neural network layer is used to fuse the above three features to enhance the signal-to-noise ratio.

9 $$\begin{array}{*{20}c} {x_{output} = Full\_Connected\left( {concat\left( {X,\hat{X},Z} \right)} \right)} \\ \end{array}$$

where $concat\left(\bullet \right)$ denotes the contact operation, and $Full\_Connected\left(\bullet \right)$ represents the calculation of a full-connected neural network layer.

Finally, after getting the representations of all nodes, we stack them in the token dimension and apply a full-connected neural network layer to compact the features.

10 $$\begin{array}{*{20}c} {Sentence\_Rep_{i} = Full\_Connected\left( {Stack\left( {x_{output1}^{i} ,{ }x_{output2}^{i} ,{ } \ldots ,x_{{output{\kern 1pt} m}}^{i} } \right)} \right)} \\ \end{array}$$

where $Stack\left(\bullet \right)$ represents the stack operation, and $Full\_Connected\left(\bullet \right)$ denotes the calculation of a full-connected neural network layer. This representation can be then used for reasoning and classification.

## Results and discussion

### Dataset and experimental settings for CPI extraction

We implemented our project in the PyTorch environment and performed evaluation using the CHEMPROT corpus. CHEMPROT [42] is a manually annotated corpus for CPI extraction, which is divided into training, development, and test sets. To concentrate on crucial CPRs, we selected 5 CPRs labeled as "Y" for evaluating our model. To ensure a fair comparison with baseline methods, the dataset used in this paper is pre-processed and provided by Peng et al. [28], and the statistics are shown in Tables 2 and 3.

**Table 2:** The statistics of CHEMPROT

	Abstracts	Annotated chemicals	Annotated proteins	Annotated CPIs
Training set	1020	13,017	12,735	4155
Development Set	612	8004	7563	2418
Test set	800	10,810	10,018	3469
Total	2432	31,813	30,316	10,042

**Table 3:** The statistics of Preprocessed CHEMPROT

	False	CPR:3	CPR:4	CPR:5	CPR:6	CPR:9
Training Set	15,306	768	2251	173	235	727
Development Set	9404	550	1094	116	199	457
Test Set	13,485	665	1661	195	293	644
Total	38,195	1983	5006	484	727	1828

We trained our model using the training set and optimized hyperparameters using the development set. The model was then trained using the optimal hyperparameters, and its performance was evaluated on the test set. The hyperparameters utilized by the proposed model are presented in Table 4.

**Table 4:** The hyper-parameters of BioEGRE

Parameters	Tuned range	Optimal
Max sequence length	128	128
Training batch size	[16, 32, 64]	64
Development batch size	8	8
Test batch size	8	8
Training epochs	50	50
Warmup proportion	0.1	0.1
Classifier dropout rate	[0.0, 0.05, 0.1]	0.0
GPNN layers	[1, 2]	1
GPNN input neighbors	[16, 20, 24, 28, 32]	32
GPNN output neighbors	[4, 8, 16]	4

In addition, considering that most CPI extraction methods use micro-averaged precision, recall, and F1-score (hereinafter referred to as Precision, Recall and F1-Score) to evaluate their models, we use the same metrics, which are defined as follows.

11 $$\begin{array}{*{20}c} {Precision_{micro} = \frac{{\sum TP_{i} }}{{\sum (TP_{i} + FP_{i} )}}} \\ \end{array}$$

12 $$\begin{array}{*{20}c} {Recall_{micro} = \frac{{\sum TP_{i} }}{{\sum (TP_{i} + FN_{i} )}}} \\ \end{array}$$

13 $$\begin{array}{*{20}c} {F1\_score_{micro} = \frac{{2{*}Precision_{micro} {*}Recall_{micro} }}{{Precision_{micro} + Recall_{micro} }}} \\ \end{array}$$

where ${TP}_{i}$ donates true positive,${FP}_{i}$ donates false positive, and ${FN}_{i}$ donates false negative for relation type $i$. Specifically, during the inferring process, it is regarded as a positive of our method that a sentence is correctly classified as its corresponding relation type.

### Performance comparison versus baseline models on the CHEMPROT corpus

In this section, we introduce the baseline models used to compare with the proposed model, and Table 5 shows the experimental results on the CHEMPROT corpus. Considering that using pre-trained LMs has become a new paradigm in NLP field, we choose pre-trained LM based methods including [5, 14, 15, 24–26, 28] as baseline models, and obtain the results from their original publications except BioELECTRA [15], PubMedBERT [24], and ClinicalBERT [25]. Because [15] misses the precision and recall. Furthermore, it is worth mentioning that the test data employed in [15, 24, 25] is reprocessed, which makes it distinct from the test data used in other baseline models and is not publicly available. Therefore, we conducted extra experiments to assess the performance of BioELECTRA, PubMedBERT, and ClinicalBERT using the same test set as [28] to ensure a fair comparison.

**Table 5:** Comparison of BioEGRE and baseline models on CHEMPROT

Methods	Precision (%)	Recall (%)	F1-score (%)
BERT + Guassian [26]	77.08	76.06	76.56
BERT + Capsule network [5]	77.78	71.86	74.70
NCBI-BERT [28]	74.5	70.6	72.5
PubMedBERT [24]	70.12	72.51	71.29
ClinicalBERT [25]	72.78	74.01	73.39
BioBERT [14]	77.02	75.90	76.46
BioELECTRA [15]	77.31	80.31	78.78
**Proposed**	**77.97**	**82.07**	**79.97**

The bold indicates list the results of the models represents the best among the results

As shown in Table 5, compared with baseline models, BioEGRE gets better precision, recall, and F1-score on CHEMPROT and increases at least 0.19% (BERT + Capsule network [5]), 1.76% (BioELECTRA [15]) and 1.19% (BioELECTRA [15]) respectively for the above metrics. Compared with BioELECTRA, BioEGRE gets a promotion of 0.66%, 1.76%, and 1.19% on precision, recall, and F1-score respectively on account of introducing topology information and using a GPNN layer. Furthermore, we conducted a case study to further explore the reasons behind the performance improvement. As depicted in Table 6, BioEGRE exhibits the ability to classify longer sentences into the correct relation types to a certain degree. This finding suggests that BioEGRE successfully captures topological features of the language, and the GPNN layer is instrumental in effectively merging local and non-local features from tokens in a sentence graph. Additionally, while BioEGRE demonstrates a slight enhancement in precision, it exhibits a noteworthy advancement in recall through the inclusion of a GPNN layer. This observation suggests that incorporating a GPNN layer effectively improves the model's capacity to capture patterns and structural features of language throughout the training process, as well as facilitates the accurate classification of sentences with the corresponding features during the testing process. Consequently, the proposed model aims to maximize the recognition of positives, thereby significantly improving recall. Nonetheless, given a constant training set, BioEGRE may struggle to acquire additional patterns and structures. Therefore, while BioEGRE successfully identifies more true positives during the inference process, it may also extract more false positives to some extent, resulting in a slight improvement in precision.

**Table 6:** Case Study for the comparison of BioEGRE and BioELECTRA

No	Sentence	Gold standard	Result from BioEGRE	Result from BioELECTRA
1	This study confirms the feasibility of using continuous measurement of AChE activity in CSF over prolonged periods, that @**rivastigmine**# markedly inhibits CSF AChE after a single oral dose of 3 mg, and that the inhibition of central AChE is substantially greater than that of peripheral AChE or @**BuChE**#	**CPR:4**	**CPR:4**	False
2	These data indicate that a @**[3H]dofetilide**# binding assay using @**HERG**# membranes may help identify compounds that prolong the QT interval	**False**	**False**	CPR:5
3	The @**GRIP1**# reduction was inhibited by @**MK-801**#, an N-methyl-d-aspartate (NMDA) receptor antagonist, but not by 6-cyano-7-nitroquinoxaline-2,3-dione (CNQX), an AMPA receptor antagonist	**CPR:4**	**CPR:4**	CPR:6

The bold represents the correct answer from the model according to gold standard

We also conduct a comparative analysis of two representative biomedical pre-trained language model, BioELECTRA and BioBERT. As shown in Table 5, BioELECTRA achieves 2.28% higher than BioBERT on F1-score. Different from BioBERT, BioELECTRA performs replaced token detection (RTD) as a pre-training task on its discriminator, making the model sensitive to both the token itself and its semantics. Given that RE tasks necessitate a high level of sensitivity towards keywords, BioELECTRA exhibits superior performance compared to BioBERT. Accordingly, we have selected BioELECTRA as the encoder for contextual features.

In summary, the proposed model outperforms existing models in terms of precision, recall, and F1-score. The results clearly indicate that integrating topological features of the language through a GPNN layer significantly enhances the performance of the model.

### The effect of different language models on performance on CHEMPROT

In this section, we perform experiments to clarify the effect of different pre-trained LMs on performance. To be specific, we utilized four alternative LMs that have demonstrated remarkable efficacy in the biomedical domain: BioBERT [14], SciBERT [23], PubMedBERT [24], and ClinicalBERT [25] as replacements for BioELECTRA to serve as encoders for contextual features. The experiments were conducted on the CHEMPROT corpus.

Table 7 depicts the performance comparison among different LMs. The experimental results indicate that LMs based on the BERT architecture exhibit a slightly lower performance compared to BioELECTRA. The above result also confirms that pre-trained LMs leveraging the generative adversarial strategy not only reduce computational costs during the pre-training phase, but also demonstrate superior performance in downstream NLP tasks that require high sensitivity to tokens/words.

**Table 7:** Performance comparison with different LMs as encoder in our method

Language model	Precision (%)	Recall (%)	F1-score (%)
BioBERT + GPNN	76.47	78.03	77.24
SciBERT + GPNN	71.86	77.78	74.70
PubMedBERT + GPNN	70.6	74.5	72.5
ClinicalBERT + GPNN	75.90	77.02	76.46
Proposed (BioELECTRA + GPNN)	**77.97**	**82.07**	**79.97**

The bold indicates list the results of the models represents the best among the results

As shown in Tables 5 and 7, an additional GPNN layer also improves the performance of BioBERT-based model, increases 2.13% and 0.78% respectively on recall and F1-score compared with BioBERT (77.08%, 76.06%, 76.56%), and the improvement of recall is noticeable. The above result demonstrates that incorporating a GPNN layer enables models to access linguistic topological features across different language models. And the prior knowledge, linguistic topology, does help models to understand the structures of language, which promotes the recall significantly.

### The effect of different parameters of GPNN layers on performance on CHEMPROT

In this section, we explore the effect of different hyper-parameters of GPNN on performance of BioEGRE, including the number of sampled nodes of GPNN $L$, the number of GPNN layers, and the number of screened out nodes of GPNN $m$. To monitor and control the variables, other parameters including training and test data are consistent with that of the reported result in Table 5.

**Fig. 4:**
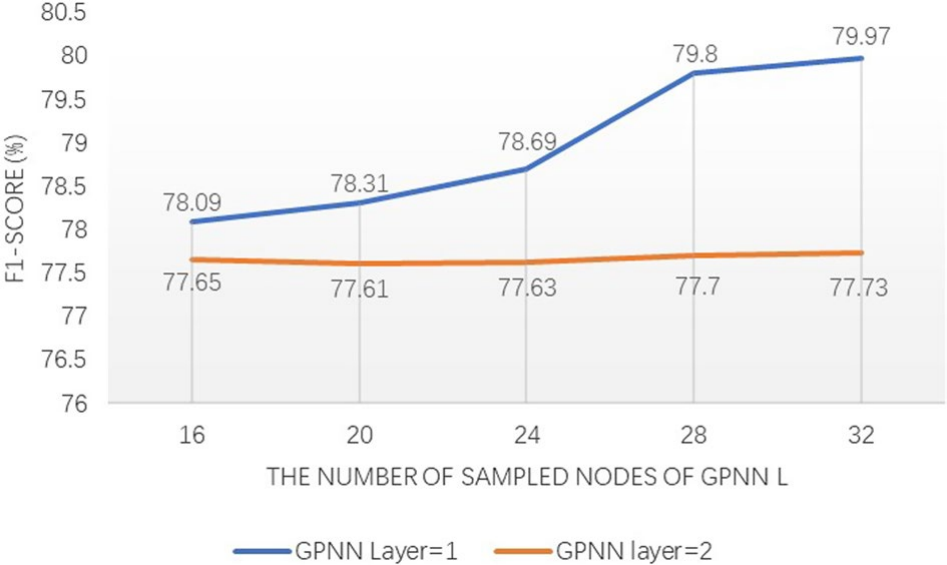
Comparison with different number of sampled nodes of GPNN

First, the effect of $L$ is analyzed. Specifically, we fix $m$ as 4, and perform experiments in the case of GPNN layers as 1 or 2, respectively. We prepare 5 optional parameters, 16, 20, 24, 28, and 32, to explore the effect of different $L$ on performance. Figure 4 illustrates the performance comparison of various $L$ of BioEGRE.

**Fig. 5:**
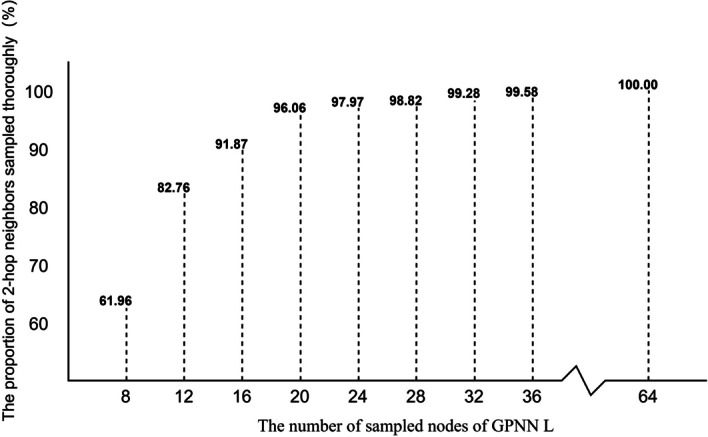
The proportion of tokens sampled completely from two-hop neighbors of nodes under different number of sampled nodes of GPNN

Experimental result shows that BioEGRE achieves the best performance when $L$ is set to 32, regardless of whether a single GPNN layer or two GPNN layers are used. Since $L$ directly affects the extent to which the neighbors of each token are sampled, we conduct a statistical analysis on tokens with fully sampled 2-hop neighbors under different $L$ values. The corresponding results are depicted in Fig. 5. When $L$ is set as 32, 2-hop neighbors of tokens can almost be sampled thoroughly, and the model achieves the best performance. Reducing $L$ may result in inadequate sampling of neighbors, leading to the omission of crucial features and consequently poor performance. Hence, it is advisable to increase the value of $L$ appropriately to ensure sufficient sampling of neighbors.

Next, we fix $L$ as 32, and change $m$ and the number of GPNN layers to explore the effect of the above two hyper-parameters on model performance. Specifically, we prepare 3 optional parameters 4, 8, 16 for $m$, and 2 optional parameters for GPNN layers 1 and 2. Table 8 shows the performance comparison for various $m$ and GPNN layers. This experimental result shows that with the same $L$, increasing the depth of GPNN layers is harmful for the performance. This may be attributed to the excessive number of parameters in a deep GPNN, which hampers effective feature extraction, subsequently resulting in a lower signal-to-noise ratio (SNR) and diminished model performance.

**Table 8:** Comparison of model effects under different GPNN parameters

	GPNN Layers = 1			GPNN Layers = 2		
	Precision (%)	Recall (%)	F1-Score (%)	Precision (%)	Recall (%)	F1-Score (%)
$m=4$	77.97	82.07	79.97	76.77	78.72	77.73
$m=8$	77.04	77.99	77.53	70.75	74.45	72.19
$m=16$	78.25	78.72	78.49	75.76	77.69	76.71

In addition, for the determination of $m$, it can be noticed that the model achieves the best performance while $m=4$ and the worst performance while $m=8$. Moreover, when $m$ is set as 16, the performance rises again. It may be because when $m$ is small ($m=4$), the model screens out the neighbors that carry the most important information for different tasks, which brings about the enhancement of SNR to generate a better-optimized distributed representation. When $m$ is large ($m=16$), the GPNN module extracts almost all the neighbors, containing all the features and some noise, and the model also works well. However, when $m=8$, the GPNN module introduces the noise and misses features to some extent, which results in the worst performance. In conclusion, the selection of $m$ should be reduced appropriately to efficiently screen out essential features and avoid noise.

### Cross validation analysis on CHEMPROT

In this section, to obtain a more robust evaluation of BioEGRE's stability, we conduct a tenfold cross-validation on the CHEMPROT dataset. To be specific, we combine the training and development sets to create a new set, and for each fold, 90% of the data in the set is utilized to train BioEGRE, while the remaining 10% is used as the development/validation set to optimize hyper-parameters. Finally, under the optimal settings, BioEGRE is evaluated on the original test set to report precision, recall, and F1-score for each fold. The performance of each fold is presented in Table 9. The experimental results demonstrate that the proposed method consistently achieves stable performance across multiple random splits of the dataset, thereby verifying the stability of BioEGRE.

**Table 9:** The result of tenfold cross-validation on the CHEMPROT dataset

Fold	Precision (%)	Recall (%)	F1-Score (%)
1	77.38	82.08	79.66
2	77.97	81.67	79.78
3	76.04	82.50	79.14
4	78.63	81.34	79.96
5	77.56	83.01	80.19
6	77.44	81.45	79.39
7	78.69	81.06	79.86
8	76.37	82.31	79.23
9	77.44	81.58	79.46
10	78.32	81.61	79.93
Ove	77.58 ± 0.83	81.86 ± 0.57	79.66 ± 0.33

### Breakdown performance analysis on CHEMPROT

To elucidate the cause of errors in BioEGRE on CHEMPROT, an analysis of the model's performance on each positive CPR type is presented in this section. Table 10 offers a breakdown of the performance on the CHEMPROT test set. Among the five CPR types, BioEGRE demonstrates exceptional performance on CPR:3, CPR:4, CPR:5, and CPR:6, while slightly underperforming on CPR:9, which aligns with the findings from BERT + Capsule network [5]. According to the investigation of Sun et al. [5], CPR:3, CPR:4, CPR:5, and CPR:6 exhibit discernible relation indicators, such as predicate verbs/phrases that reveal the specific relationship in sentences, while CPR:9 lacks apparent indicators. When a sentence contains explicit relation indicators, these features are more likely to be captured by the GPNN module at the linguistic topological level, contributing to more accurate predictions. However, without an apparent indicator, the GPNN module plays a less obvious role, resulting in poor performance on such CPR type.

**Table 10:** Breakdown performance on CHEMPROT corpus

CPR type	Precision (%)		Recall (%)		F1-Score (%)	
	BERT + Capsule network [5]	BioEGRE	BERT + Capsule network [5]	BioEGRE	BERT + Capsule network [5]	BioEGRE
CPR:3	78.61	76.76	67.97	81.95	72.90	79.27
CPR:4	78.03	80.31	79.11	90.13	78.57	84.94
CPR:5	77.78	74.65	68.21	81.54	72.68	77.94
CPR:6	81.41	77.13	74.74	86.35	77.94	81.48
CPR:9	73.99	73.14	56.99	59.63	64.39	65.70

Table 11 shows the confusion matrix on the CHEMPROT test set. The rows denote the predictions of BioEGRE, and the columns represent the gold-standard annotations. From Table 11, it can be inferred that BioEGRE basically distinguishes positive CPRs, and that the model confuses sentences marked as positive CPRs with those marked as False. To our knowledge, Peng et al. [28] marks not only sentences with non-evaluated CPR types, but also manually constructed negative samples without any association with CPRs, as False during preprocessing. Moreover, a significant portion of False samples comprises the latter category. During the inferring process, BioEGRE may mistakenly classify some sentences with non-evaluated CPR types as positive, leading to incorrect predictions. Therefore, we can deduce that the primary challenge in the CPI extraction task lies in distinguishing between non-evaluated and evaluated CPR types, as well as extracting common features from sentences with non-evaluated relation types and manually constructed negative samples.

**Table 11:** The confusion matrix on the CHEMPROT test set

Prediction	Gold-standard					
	False	CPR:3	CPR:4	CPR:5	CPR:6	CPR:9
False	12,720	101	146	34	39	253
CPR:3	133	545	9	2	0	1
CPR:4	247	14	1497	0	1	5
CPR:5	50	3	0	159	0	1
CPR:6	72	0	3	0	253	0
CPR:9	253	2	6	0	0	384

### Experimental results on the GAD and EU-ADR corpus

To evaluate the generalizability of BioEGRE, we conducted experiments on other biomedical RE tasks. Specifically, BioEGRE is trained and tested on the Gene Association Database (GAD) [44], which is labeled with gene-disease relations, and EU-ADR [45], which is labeled with disease-target relations.

The GAD corpus is oriented to the correlation between genes and diseases in genetic association studies, containing 10,697 genes, 12,774 diseases, and 74,928 gene-disease relations (labeled as positive). In addition, the EU-ADR corpus focuses on the correlation between diseases and targets in scientific literature abstracts, which contains 7,011 annotated entities and 2,436 relations.

The above two biomedical RE tasks can be formulated into binary classification problems, and the datasets on which BioEGRE is trained and tested are preprocessed by Lee et al. [14]. Meanwhile, we use BioBERT [14] and BioELECTRA [15] as baseline methods. We replicated the baselines and utilized precision, recall, and F1-score as evaluation metrics. The performance comparison between BioEGRE and the baseline methods is presented in Table 12. Additionally, to assess the stability of our proposed model, we conducted a tenfold cross-validation on the GAD and EU-ADR datasets. The results are summarized in Table 13, where p, r, and f represent precision, recall, and F1-score respectively, and Ove. denotes the overall performance (mean ± std).

**Table 12:** Performance comparison with baseline models on GAD and EU-ADR

Models	GAD			EU-ADR		
	p (%)	r (%)	f (%)	p (%)	r (%)	f (%)
BioBERT [14]	77.32	82.68	79.83	77.86	83.55	79.74
BioELECTRA [15]	78.15	84.29	81.10	73.33	81.48	77.19
**Proposed**	**79.77**	**87.20**	**83.31**	**81.73**	**85.37**	**83.51**

The bold indicates list the results of the models represents the best among the results

**Table 13:** Cross-validation performance of BioEGRE on GAD and EU-ADR

Fold	GAD			EU-ADR		
	p (%)	r (%)	f (%)	p (%)	r (%)	f (%)
1	79.55	87.19	83.19	81.48	84.62	83.02
2	79.21	86.71	82.79	81.84	83.33	82.58
3	79.33	87.30	83.12	80.67	85.11	82.83
4	78.97	86.67	82.64	81.48	84.52	82.97
5	79.75	87.21	83.31	81.75	85.34	83.51
6	79.33	87.67	83.29	81.98	84.76	83.35
7	78.82	88.11	83.21	80.45	84.36	82.36
8	79.01	87.42	83.00	80.10	84.57	82.27
9	80.01	86.33	83.05	81.33	90.15	85.51
10	78.21	86.52	82.16	80.36	83.02	81.67
Ove	79.21 ± 0.51	87.11 ± 0.55	82.98 ± 0.36	81.14 ± 0.68	84.98 ± 1.95	83.00 ± 1.03

As shown in Table 12, BioEGRE gets the best performance in the above biomedical RE tasks compared with baseline methods, which demonstrates the generalization of BioEGRE. Additionally, as shown in Table 13, BioEGRE also has a strong stability. However, the result in the ninth fold in EU-ADR corpus is prominent. We delved into the data of the ninth fold and discovered that it contains a higher proportion of positive instances compared to other folds. This disparity may explain the exceptional performance observed. In conclusion, the experimental results affirm the generalization and stability of the proposed method.

### Discussion and limitation

The model achieves better performance mainly for the following reasons. (1) A graph instead of a sequence is used to model the topology of a sentence, which can help to incorporate the topological knowledge. (2) BioELECTRA is used to encode the contextual features and a GPNN layer is utilized to optimize the distributed representation, which not only captures contextual features effectively but also captures both local and non-local features within the sentence graph, enabling the generation of a more accurate sentence-level distributed representation.

As for the generalization, the proposed method is able to be extended to general domains and other RE tasks rather than limited to the biomedical field. First, BioRE task is more complicated, which is because biomedical texts contain more obscure words, and appear in the form of long and difficult sentences. Secondly, BioEGRE not only exhibits excellent performance in multi-class relation extraction tasks, such as CPI extraction, but also demonstrates effective results in binary relation extraction tasks focused on GAD and EU-ADR, which indicates the potential for BioEGRE to be theoretically generalized and applied to diverse relation extraction tasks across various fields.

When it comes to the processing time of BioEGRE, we have conducted experiments to test the speed of our model in the training process and inferring process. The experimental environment is a server with a 24-core, Inter® Xeon® Gold 6248R CPU, 3.0 GHz-frequency, a single A100 PCIE 40 GB GPU and 512 GB-memory. The operating system is 64-bit Ubuntu 16.04.4 LTS (GNU / Linux 4.13.0–36 -generic x86_64). Furthermore, we have performed the test on the CHEMPROT dataset, with epochs as 50, max sequence length as 128, batch size as 64, the number of sampled nodes as 32, the number of GPNN layers as 1, and the number of screened out nodes as 4. As for the training process, the model cost about 180 min (BioELECTRA cost about 120 min with the same hyper-parameters), where it took 15 min to preprocess the data, and 165 min to train the neural network parameters of the model, including fine-tuning BioELECTRA and training parameters of GPNN and full-connected neural network. Despite the additional time required (an additional 60 min for 50 epochs compared to BioELECTRA) and computational cost associated with the parsing process and GPNN module, the substantial improvement in performance, resulting from the inclusion of topological features, justifies this tolerable overhead.

It is also important to acknowledge the limitations of the proposed model. Firstly, while SciSpaCy is considered one of the top tools for parsing biomedical texts, it is not infallible and may occasionally produce inaccurate parsing results. Consequently, the use of SciSpaCy in our model may introduce some noise and potentially lead to errors in our predictions. To mitigate this issue, we suggest exploring a multi-task learning strategy that combines dependency parsing and RE simultaneously, which could potentially enhance the overall performance of our model. Second, BioEGRE is essentially a pipeline model for RE tasks, which is only oriented to manually tagged sentences. However, pipeline models may only account for sentence-level information and fail to fully utilize the entity-level features of a sentence. Consequently, we suggest that exploring a multi-task learning strategy that jointly involves NER and RE tasks could be highly beneficial in improving the performance of BioEGRE. Third, there exists some versions of pre-processed data of CHEMPROT, bringing about the incomparability of the results of methods based on different pre-processed data. For instance, SciBERT [23] and BioM-BERT [46] achieve F1-score of over 80% on a pre-processed CHEMPROT dataset different from that provided by Peng et al. [28], while [15] reports that SciBERT achieves 75.24% of F1-score on the same dataset to evaluate BioEGRE. This inconsistency highlights the need for a unified and standardized pre-processed dataset for CHEMPROT to enable unbiased evaluations of BioRE methods.

## Conclusion

Automatic and accurate extraction of relations from biomedical literature plays a significant role in biomedical natural language processing (NLP). In this paper, we propose a novel approach, BioEGRE, for sentence-level BioRE. Different from existing methods, BioEGRE incorporates linguistic topological features and leverages a GPNN layer to effectively merge local and non-local features of tokens. The experimental results demonstrate that BioEGRE outperforms the baseline methods on the CHEMPROT, ADE, and GAD corpora, indicating the effectiveness and generalizability of the proposed method. In the future, our research direction involves integrating a multi-task learning strategy that combines dependency parsing and NER with the RE task. Moreover, we plan to incorporate GPT-based generative models to further enhance the performance of our approach.

## Data Availability

We make the source code and model available at https://github.com/zxw1995shawn/BioEGRE.

## Abbreviations

BFS: Breath first search
BioRE: Biomedical relation extraction
CPI: Chemical–protein interaction
CPR: Chemical–protein relation
GAT: Graph attention network
GCN: Graph convolutional network
GNN: Graph neural network
GPNN: Graph pointer neural network
NLP: Natural language processing
RE: Relation extraction
RTD: Replaced token detection
SNR: Signal-to-noise ratio
SOTA: State of the art
